# Contactless monitoring to prevent self-harm and suicide in custodial settings: Protocol for a global scoping review

**DOI:** 10.1136/bmjopen-2024-087925

**Published:** 2024-10-26

**Authors:** Rebecca Bosworth, Bronwyn Everett, Paul Breen, Jason Klein, Eleni Psillakis, Penelope Abbott, Kirsty Smith, Wanqing Li, Neil Anderson, Chetan Singh Thakur, Rohan Borschmann

**Affiliations:** 1 School of Nursing, Faculty of Science, Medicine and Health, University of Wollongong, Wollongong, New South Wales, Australia; 2 National Drug and Alcohol Research Centre, University of New South Wales Faculty of Medicine, Sydney, New South Wales, Australia; 3 The MARCS Institute, Western Sydney University, Penrith, New South Wales, Australia; 4 NSW Police Service, Parramatta, New South Wales, Australia; 5 Justice Health and Forensic Mental Health Network, Sydney, New South Wales, Australia; 6 School of Medicine, Western Sydney University, Sydney, New South Wales, Australia; 7 School of Computing and Information Technology, Faculty of Engineering and Information Sciences, University of Wollongong, Wollongong, New South Wales, Australia; 8 3 AIM Solutions, Sydney, New South Wales, Australia; 9 Indian Institute of Science, Bangalore, India; 10 Murdoch Childrens Research Institute, Parkville, Victoria, Australia; 11 Department of Psychiatry, Medical Sciences Division, University of Oxford, Oxford, UK

**Keywords:** Suicide and self-harm, Prisons, PUBLIC HEALTH

## Abstract

**Abstract:**

**Introduction:**

Self-harm and suicide are major contributors to the global burden of disease and people in custodial settings are at a markedly increased risk of these adverse outcomes. Contactless monitoring technology is emerging as a possible solution to prevent self-harm and suicide by detecting and predicting vulnerabilities among people at increased risk in custodial settings in realtime, however no reviews to date have synthesized the evidence base, in the custodial context, regarding (a) the extent to which this technology has been implemented; and (b) the acceptability and feasibility of its application among custodial staff, specifically in relation to maintaining the wellbeing and safety of both incarcerated people and custodial professionals.

**Methods and analysis:**

Our scoping review will be reported in accordance with the Preferred Reporting Items for Systematic reviews and Meta-Analyses extension for Scoping Reviews guidelines. We searched key electronic health and social science databases (MEDLINE, PubMed, Scopus, Web of Science, ProQuest and Google Scholar) on 5 February 2024 for peer-reviewed studies, which report on the use of contactless monitoring in custodial settings. Any type of study design was eligible, and the publication format was not limited. We included quantitative peer-reviewed journal articles, all types of reviews (narrative, systematic and meta-analysis) and did not apply study eligibility restrictions on country of origin. We will also search grey literature. Inclusion of publications will be restricted to the English language.

**Ethics and dissemination:**

This review does not require institutional ethics review or approval as it is a review of studies that have already been granted relevant ethics approval. Our dissemination strategy includes a peer-reviewed publication and presentations at relevant national and international academic conferences. A plain language summary will be distributed through consumers and professional networks.

**STRENGTHS AND LIMITATIONS OF THIS STUDY**

- This scoping review uses a comprehensive search strategy including six academic databases.
- Our review is further strengthened by the inclusion of grey literature, enhancing the comprehensiveness of findings.
- An additional strength is the diverse background of the research team, comprising expert by lived experience of incarceration and mental ill-health, academics, clinicians, managers, stakeholders and industry professionals with expertise in various fields including psychology, nursing, engineering, police, medicine, justice health and forensic mental health.
- A limitation of this study is that correctional officers are not included in the team of stakeholders.
- Another limitation of this study is that this review will be limited to publications only in the English language.

## Introduction

 Self-harm among incarcerated people is a significant public health concern globally,^1 2^ with an estimated prevalence of 5%–6% among men and 20%–24% among women incarcerated in the UK.^1^ The most serious adverse outcome associated with self-harm in prison is suicide^3^ and, in 2020, the annual rate of suicide among incarcerated males with a history of self-harm in England and Wales was 334 per 100 000, compared with 95 per 100 000 for incarcerated individuals with no history of self-harm.^3^ Adolescents (ie, those aged 10–24 years)^4^ detained under the criminal justice system are also at an increased risk of suicidal behaviours when compared with their age-and sex-matched peers in the general population.^5^ In 2020, suicide was the most common cause of death among incarcerated people in Europe, followed by COVID-19 and drug overdose.^6^ In Australia, 5.1% of people discharged from prison reported self-harm during the current period of incarceration in 2022.^7^ In 2021–2022, of the 63 deaths in Australian prisons where the manner of death was recorded, 25% were from death by suicide or self-inflicted and 22% were death by hanging.^7^ The burden of self-harm among people in prison is likely to increase further, given the global imprisonment rate is also increasing annually, and importantly, this rate is increasing at a faster rate than population growth.^1 8 9^ The health profiles of people in custody are often complex, with many living with co-occurring conditions;^6^ for example, approximately 50% of the global prison population living with non-affective psychosis or major depression are also living with a comorbid substance use disorder.^10^ This further increases the risk of other potentially fatal medical episodes during custody, particularly intoxication and overdose.^11^

Currently, a range of monitoring procedures are used in custodial settings to minimise adverse outcomes associated with self-harm, suicide attempts and alcohol and other substance use,^12^ as well as potential complications following the use of restraint and the administration of medications.^13^ Traditional monitoring methods include risk assessments and visual observations, both physically and remotely, yet key differences exist. Risk assessments are informed by both clinical intuition and screening on entry into custody and as circumstances or conditions change,^14^,^16^ assessing for signs of intoxication and/or withdrawal, consciousness levels, head injuries, substance concealment and self-harm or non-fatal suicide attempt history.^11^ Risk assessments are an ongoing process and refer to structured, point-in-time evaluations used to determine whether increased monitoring such as continuous observation or checks at specific intervals are indicated.^14^ Risk assessments are also used to determine if monitoring can be decreased, indicated by the individual’s clinical condition and future risk factors for suicide or self-harm.^17^ Visual observations refer to watching for observable changes that may indicate risk and may include physically checking for signs of life via positional changes, attempts to rouse when sleeping or movement in the rise and fall of the chest,^11 13 16^ either from the cell door or remotely via closed-circuit teleision (CCTV) video surveillance.^11 14 15^ Monitoring vital signs (eg, heart rate, blood pressure, respiratory rate and temperature) is often beyond the scope of non-medical custodial staff and therefore dependent on the presence and input of health professionals.

Unlike traditional intrusive methods, contactless monitoring uses contactless techniques and, depending on the application, different technologies may be used to remotely and continuously monitor vital signs and evaluate risk factors and behaviours.^18^,^20^ These include radar-based systems, machine learning, edge computing (processing data closer to a collection point, to act on data and provide faster insights in real-time)^19 21^ and image- and non-image-based systems.^22^ Contactless monitoring technology has evolved markedly in the last decade,^18^ boasting a broad array of potential application scenarios spanning not only the corrective services sector^22 23^ but also national security, aged care, MedTech, home care, livestock, veterinary services and healthcare.^23^ Contactless monitoring technology is emerging as a solution to supplement current monitoring procedures custodial staff use to monitor the well-being of people at increased risk while in their cells.^12 14^ By monitoring vital signs, risk factors and behaviours, any deviations that could signal an impending health event can raise an alarm in real-time and alert the custodial staff, who can then intervene. The implementation of the technology, in combination with existing methods, may overcome some of the limitations of traditional monitoring. Limitations may include the reliance on physical visual checks conducted through spy holes, flaps in the door or perspex panels, the misinterpretation of life-threatening conditions for drug or alcohol consumption,^14^ outdated technology affecting CCTV quality and blind spots, complications between scheduled checks,^13^ ethical privacy concerns and over-reliance on these methods to the exclusion of others.^24^

While contactless monitoring technology shows promise for addressing critical health concerns in custodial settings, its development stage must be considered. Despite advancements, contact-based sensors remain the norm in healthcare. Contactless monitoring is not yet a standard practice, with limited literature on its effectiveness on acutely unwell or deteriorating patients.^25^ Validation against gold standard measurements in traditional settings is unclear.^25^ Both radar- and camera-based techniques face challenges affecting accuracy and applicability.^26^ A review by Khanam *et al* (2019)^26^ on remote monitoring of vital signs in diverse non-clinical and clinical scenarios using computer vision systems provides an assessment of image-based monitoring and highlights some deficiencies, including (1) automatic selection of multiple regions of interest, (2) noise and motion artefact removal, (3) simultaneous multiperson monitoring, (4) long-distance detection, (5) multicamera fusion, (6) low lighting conditions and (7) the lack of publicly available datasets from realistic scenarios.^26^ Wireless video-based patient monitoring was reviewed in a systematic review by Harford *et al* (2017),^27^ identifying several significant shortcomings including (1) minimal testing or validation in clinical settings, (2) a predominant focus on neonates rather than children or adults and (3) inadequate data for validation in laboratory settings, particularly concerning the duration of testing and the range of vital signs assessed in healthy participants. Radar-based technologies also encounter issues such as body movement interference and the lack of efficient and stable signal processing techniques capable of handling low sample data.^28^ While Doppler radar has shown feasibility for vital sign monitoring in controlled environments, additional work is needed to improve signal quality analysis for better breathing and heart rate estimation.^24^ In prison settings, additional sources of motion like ceiling fans and water movement from sinks and toilets flushing further affect radar signal quality and increase false alarms.^12 24^ Thus, challenges remain to widespread adoption in clinical settings and necessitate further research.

Contactless monitoring technologies, while promising, often show reduced accuracy and reliability compared with traditional methods. However, studies have shown Doppler radar can match wearable device outputs within +/−5% for heart and respiratory rates.^12^ Gupta (2022)^29^ reported 93.2%–100% accuracy for medical radar compared with contact-type ECGs and respiration belts. Camera-based measurements also perform well under ideal conditions but highlight performance variability.^29 30^ When considering the implementation of contactless monitoring technology, it is important to note its current deployment and acceptance in clinical settings. Some areas, like neonatal intensive care units, use camera imaging-based systems using imaging photoplethysmography (iPPG) for continuous monitoring, including heart rate, respiratory rate, skin temperature and oxygen saturation.^31 32^ iPPG has also been used for patients undergoing haemodialysis.^33 34^ Trials at the Royal Melbourne Hospital involve radar imaging and thermal scanners for rapid assessment in older patients.^35^ A review by Grech (2024)^36^ reported on 15 hospital-based studies on non-contact red-green-blue camera-based heart rate and rhythm monitoring in adult clinical settings, including emergency departments, postoperative care units, general medical wards and haemodialysis units. However, the review highlights ongoing challenges with patient movement, illumination and technique standardisation that must be overcome for widespread adoption.^36^

Limited literature exists on staff perceptions of contactless monitoring. Ede *et al* (2021)^37^ explored intensive care unit staff expectations, finding the concept acceptable with perceived usability benefits for both patients and staff. Contactless monitoring may offer a sustainable solution, yet staff need to be comfortable and familiar with the system and able to troubleshoot issues independently.^37^ Nevertheless, perceived acceptability does not equate to proven reliability or accuracy of the technology. Despite the promise of this technology, the continuous collection of sensor data in healthcare settings presents significant ethical concerns about privacy, data management, bias, fairness and informed consent.^38^ Therefore, addressing these issues is crucial to identify and mitigate potential harms, ensuring transparency and accountability and building trustworthy and ethically sound systems.^38^

Contactless monitoring technology is considered to hold particular significance in addressing critical health concerns such as self-harm, suicide and substance use in custodial settings.^39^ However, no reviews to date have synthesized the evidence base, in the custodial context, regarding the feasibility and acceptability from the perspective of end users, including people detained in custodial settings, custodial officers and healthcare staff, and on the extent to which contactless monitoring has been implemented in custodial settings. In this context, we have produced a robust protocol for the first scoping review globally to synthesize research relating to: (a) the contactless monitoring technologies implemented in custodial settings; (b) the benefits, limitations and/or challenges of implementing contactless monitoring in custodial settings and (c) the knowledge, attitudes and/or perceptions among custodial and healthcare staff towards contactless monitoring, specifically in relation to maintaining the well-being and safety of both people in custodial settings and staff. It must be acknowledged that this scoping review aims to map the existing literature rather than provide a prevalence estimate. Therefore, the prevalence of existing studies should not be confused with the prevalence of the technologies used.

## Methods and analysis

### Protocol and registration

This scoping review protocol was developed according to the Joanna Briggs Institute (JBI) guidelines^40^ and the review will adopt the approach of Arksey and O’Malley’s framework.^41^ We will follow the Preferred Reporting Items for Systematic Reviews and Meta-Analysis (PRISMA) protocols and apply the PRISMA extension for scoping reviews to present the results^42^ (online supplemental 1).

### Search strategy and information sources

We searched six key electronic health, social science and engineering databases (MEDLINE, PubMed, Scopus, Web of Science and ProQuest and Google Scholar was searched via Harzings Publish or Perish) for studies published in English, which report on the use of contactless monitoring in custodial settings. Publication date was limited from database inception to 5 February 2024. We used variants and combinations of search terms related to contactless vital sign monitoring, proof of life, police, custody, prison, jail, self-harm and suicide. The search strategy was developed in consultation with a research librarian from the faculty of Science, Medicine and Health at the University of Wollongong, Australia, to ensure optimal rigour (online supplemental 2). To locate additional relevant publications not identified during the database searches, reference lists of published review articles will be scrutinised. We will search websites of relevant authorities, organisations and stakeholders (eg, the International Corrections and Prisons Association),^43^ the International Association of Chiefs of Police and the Global Law Enforcement and Public Health Association^44^ for any additional literature, including conference websites for relevant abstracts (eg, innovative technology conferences, law enforcement technology, police technology forums/conferences and corrections technology conferences). Publication format will not be limited and will include qualitative and quantitative peer-reviewed journal articles, including all types of review (narrative, systematic and meta-analysis) as well as grey literature. Any type of study design was eligible (eg, randomised controlled trials, case-control studies, prospective or retrospective cohort studies, quasi-experimental and qualitative), publication format was not limited, and no study eligibility restrictions on country of origin were applied. Inclusion and exclusion criteria are presented in online supplemental 3).

### Study selection

Publication details and abstracts for each of the studies identified through the electronic search were imported into EndNote21 reference management software^45^ and then transferred into Covidence,^46^ before duplicates were removed. Study selection will consist of two stages of screening: (1) title and abstract review and (2) full-text review. During the first stage, the titles and abstracts of all remaining studies will be independently screened by Reb B, BE, and JK against specified inclusion/exclusion criteria for potential inclusion (online supplemental 3). PE will resolve any conflicts. Roh B will be involved in the rescreening of 10% of studies included in the initial screen (title and abstract) to test the validity of the approach and discuss potential alterations. During the second stage, full-text reviews of the remaining publications will be conducted independently by Reb B and BE, and reference lists of potentially relevant publications will be manually searched against specified inclusion/exclusion criteria to locate additional references. Forward searching will also be conducted.^47^ Double screening will be conducted during all stages of the review. Uncertainty regarding whether publications met the inclusion criteria will be resolved through discussion among members of the team. In instances when the full text of potentially relevant publications cannot be located, a maximum of two attempts will be made to contact the author(s) via email to request a copy. The JBI Critical Appraisal Checklist will be used to assess the methodological quality of all primary research publications by evaluating the extent to which they addressed the possibility of bias in nine areas of study design, conduct and analysis.^48^ Scoping reviews aim to provide an overview of evidence, rather than produce a critically appraised and synthesised result; therefore, an assessment of methodological limitations or risk of bias is generally not performed.^49^ Included studies can be on any of (a) contactless monitoring technologies implemented in custodial settings; (b) benefits, limitations and/or challenges of implementing contactless monitoring in custodial settings and/or (c) knowledge, attitudes and/or perceptions of technology among custodial staff.

### Data extraction

Data from all final included full-text articles will be extracted by two or more independent reviewers using a data extraction tool developed by the authors. Any disagreements that arise between the reviewers will be resolved through discussion, or with an additional reviewer(s). A standardised Excel template will be used to extract data which will include, yet will not be limited to, details regarding author(s) and year of publication, country, study design, setting, participant characteristics, types of contactless monitoring implemented, knowledge/attitudes/perceptions of custodial and healthcare staff towards the feasibility and acceptability of the technology and relevant evaluation and outcome data, such as contactless monitoring trial evaluations and/or effectiveness/outcomes of currently implemented technologies.

Text and tables will be used to collate, summarise and report the extracted data. Following the extraction of relevant information, content analysis will be conducted on the extracted data. The data extraction form will be refined and updated throughout the review based on emergent findings, given a scoping review is an iterative process. Modifications will be detailed in the scoping review. Online supplemental 4 outlines the preliminary data extraction plan for this scoping review.

### Collating, summarising and reporting the results

Given a scoping review can be used to map the concepts underpinning a research area and the main sources and types of evidence available, a narrative summary will accompany the tabulated and/or charted results. The summary will provide an overview of the research and describe how the results relate to the scoping review’s objective and question(s), rather than an assessment of the quality of individual studies.

### Risk of bias assessment

Scoping reviews do not typically include a risk of bias assessment;^40^ therefore, this assessment was not conducted.

### Patient and public involvement

In accordance with the BMJ guidance on patient and public involvement statement,^50^ public partners were engaged in this study. A public partner with lived experience of incarceration and mental ill health is involved in the design, conduct, reporting or dissemination plans of this research. First, contact was sought to seek interest in collaborating with the team on the review, via email and a follow-up phone conversation to provide background and context. The public partner was advised to assess their interest after being advised of the potential burden involved. This includes helping the researchers review the first draft and provide insight and feedback, as well as continuing to contribute to edits of the paper and be a co-author. Following consent, the member was provided with the first draft via email, and permission was sought to follow-up with an online Zoom meeting to discuss any recommendations or feedback and allow for active contribution. The protocol and research questions were drafted prior to engagement with the public partner, with the view to amend as required as engagement was not sought prior to this point. Following review, based on the public partner’s experience of incarceration, a curiosity emerged as to the availability of the technology. The public partner shared their perceived benefits and limitations on the technology as well as the perceived acceptability by custodial staff. Therefore, the draft research questions aligned with the public partner’s experience and areas of interest. The priorities and preferences of the public partner in relation to the topic were shared with the view of integrating ideas into the discussion of the review. The public partner and research team will be involved in dissemination. The team will be disseminating plain language findings to consumer organisations with an interest in the health of incarcerated individuals.

### Ethics and dissemination

This review does not require institutional ethics review or approval as this is a review of studies that have already been granted relevant ethics approval. Our dissemination strategy includes a peer-reviewed academic publication and presentations at relevant national and international conferences. A plain language summary will be distributed through the authors’ and professional networks.

## supplementary material

10.1136/bmjopen-2024-087925 online supplemental file 1
