# Supplementary material for: Contactless monitoring to prevent self-harm and suicide in custodial settings: Protocol for a global scoping review
Source: BMJ Open. 2024 Oct 26;14(10):e087925. doi: 10.1136/bmjopen-2024-087925 (PMC11529512; doi:10.1136/bmjopen-2024-087925)
Supplement: online supplemental file 1 [file bmjopen-14-10-s001.pdf]

Supplementary files

Supplementary 1 (S1)

Table 1: PRISMA-ScR Checklist

**SECTION**	**ITEM**	**PRISMA-SCR CHECKLIST ITEM**	**REPORTED ON PAGE #**
**TITLE**			
Title	**1**	*Identify the report as a scoping review.*	1
**ABSTRACT**			
Structured summary	**2**	*Provide a structured summary that includes (as applicable): background, objectives, eligibility criteria, sources of evidence, charting methods, results, and conclusions that relate to the review questions and objectives.*	2
**INTRODUCTION**			
Rationale	**3**	*Describe the rationale for the review in the context of what is already known. Explain why the review questions/objectives lend themselves to a scoping review approach.*	3-5
Objectives	**4**	*Provide an explicit statement of the questions and objectives being addressed with reference to their key elements (e.g., population or participants, concepts, and context) or other relevant key elements used to conceptualize the review questions and/or objectives.*	5
**METHODS**			
Protocol and Registration	**5**	*Indicate whether a review protocol exists; state if and where it can be accessed (e.g., a Web address); and if available, provide registration information,*	N/A for protocol

		including the registration number.	
Eligibility criteria	**6**	*Specify characteristics of the sources of evidence used as eligibility criteria (e.g., years considered, language, and publication status), and provide a rationale.*	6, Supplementary 3
*Information sources**	**7**	*Describe all information sources in the search (e.g., databases with dates of coverage and contact with authors to identify additional sources), as well as the date the most recent search was executed.*	6
Search	**8**	*Present the full electronic search strategy for at least 1 database, including any limits used, such that it could be repeated.*	Supplementary 2
Selection of sources of evidence†	**9**	*State the process for selecting sources of evidence (i.e., screening and eligibility) included in the scoping review.*	6-7
Data charting process‡	**10**	*Describe the methods of charting data from the included sources of evidence (e.g., calibrated forms or forms that have been tested by the team before their use, and whether data charting was done independently or in duplicate) and any processes for obtaining and confirming data from investigators.*	7
Data items	**11**	*List and define all variables for which data were sought and any assumptions and simplifications made.*	Supplementary 4
Critical appraisal of individual sources of evidence§	**12**	*If done, provide a rationale for conducting a critical appraisal of included sources of evidence; describe the*	N/A

		methods used and how this information was used in any data synthesis (if appropriate).	
Synthesis of results	**13**	*Describe the methods of handling and summarizing the data that were charted.*	7
**RESULTS**			
Selection of sources of evidence	**14**	*Give numbers of sources of evidence screened, assessed for eligibility, and included in the review, with reasons for exclusions at each stage, ideally using a flow diagram.*	N/A for protocol
Characteristics of sources of evidence	**15**	*For each source of evidence, present characteristics for which data were charted and provide the citations.*	N/A for protocol
Critical appraisal within sources of evidence	**16**	*If done, present data on critical appraisal of included sources of evidence (see item 12).*	N/A
Results of individual sources of evidence	**17**	*For each included source of evidence, present the relevant data that were charted that relate to the review questions and objectives.*	N/A for protocol
Synthesis of results	**18**	*Summarize and/or present the charting results as they relate to the review questions and objectives.*	N/A for protocol
**DISCUSSION**			
Summary of evidence	**19**	*Summarize the main results (including an overview of concepts, themes, and types of evidence available), link to the review questions and objectives, and consider the relevance to key groups.*	N/A for protocol
Limitations	**20**	*Discuss the limitations of the scoping review process.*	N/A for protocol
Conclusions	**21**	*Provide a general interpretation of the results with respect to the review questions and*	N/A for protocol

		objectives, as well as potential implications and/or next steps.	
**FUNDING**			
	**22**	*Describe sources of funding for the included sources of evidence, as well as sources of funding for the scoping review. Describe the role of the funders of the scoping review.*	8

Source: Tricco AC, Lillie E, Zarin W, O'Brien KK, Colquhoun H, Levac D, et al. PRISMA Extension for Scoping Reviews (PRISMA- ScR): Checklist and Explanation. Ann Intern Med;169:467–473. doi: 10.7326/M18-0850 (41)

Supplementary 2 (S2)

Table 2: Database search strategies

Database	Search strategy
Medline	AB (prison* OR prisoner OR inmate* OR "prison escort" OR detainee OR detention OR jail OR gaol OR "secure unit" OR "holding cell" OR "solitary confinement" OR captivity OR internment OR incarceration OR "police custody" OR custody OR correctional) AND AB ("non-contact vital sign monitor" OR "contactless AND monitoring" OR "vital sign monitoring" OR "Life Sign Monitoring System" OR "Contact-free Sensing" OR "proof of life" OR breathing OR "biological signal") OR TX (prison* OR prisoner OR inmate* OR "prison escort" OR detainee OR detention OR jail OR gaol OR "secure unit" OR "holding cell" OR "solitary confinement" OR captivity OR internment OR incarceration OR "police custody" OR custody OR correctional) AND TX ("non-contact vital sign monitor" OR "contactless AND monitoring" OR "vital sign monitoring" OR "Life Sign Monitoring System" OR "Contact-free Sensing" OR "proof of life" OR breathing OR "biological signal")
Web of Science	prison* OR prisoner OR inmate* OR "prison escort" OR detainee OR detention OR jail OR gaol OR "secure unit" OR "holding cell" OR "solitary confinement" OR captivity OR internment OR incarceration OR "police custody" OR custody OR correctional (Topic) AND (non-contact vital sign monitor) OR "contactless AND monitoring" OR "vital sign monitoring" OR "Life Sign Monitoring System" OR (Contact-free Sensing) OR (proof of life) OR breathing OR "biological signal" (Topic)
ProQuest	title(prison* OR prisoner OR inmate* OR "prison escort" OR detainee OR detention OR jail OR gaol OR "secure unit" OR "holding cell" OR "solitary confinement" OR captivity OR internment OR incarceration OR "police custody" OR custody OR correctional) AND title(non-contact vital sign monitor OR "contactless AND monitoring" OR "vital sign monitoring" OR "Life Sign Monitoring System" OR Contact-free Sensing OR proof of life OR breathing OR "biological signal") AND summary(prison* OR prisoner OR inmate* OR "prison escort" OR detainee OR detention OR jail OR gaol OR "secure unit" OR "holding cell" OR "solitary confinement" OR captivity OR internment OR incarceration OR "police custody" OR custody OR correctional) AND summary(non-contact vital sign monitor OR "contactless AND monitoring" OR "vital sign monitoring" OR "Life Sign Monitoring System" OR Contact-free Sensing OR proof of life OR breathing OR "biological signal")

PubMed	(prison* OR prisoner OR inmate* OR "prison escort" OR detainee OR detention OR jail OR gaol OR "secure unit" OR "holding cell" OR "solitary confinement" OR captivity OR internment OR incarceration OR "police custody" OR custody OR correctional) AND (non-contact vital sign monitor "contactless AND monitoring" "vital sign monitoring" OR "Life Sign Monitoring System" Contact-free Sensing OR proof of life OR breathing OR "biological signal")
Scopus	(TITLE-ABS-KEY<br>(prison* OR prisoner or inmate* OR "prison escort" OR detainee OR detention OR jail OR gaol OR "secure unit" OR "holding cell" OR "solitary confinement" OR captivity OR internment OR incarceration OR "police custody" OR custody OR correctional) AND TITLE-ABS-KEY (OR "contactless and monitoring" OR "vital sign monitoring" OR "life sign monitoring system" OR {contact-free sensing} OR {proof of life} OR breathing OR "biological signal")
Google Scholar (Harzings via Publish or Perish)	vital sign monitoring prison police prison OR gaol OR jail OR cell OR police OR custody OR holding OR detention OR centre "contactless"

Supplementary 3 (S3)

Table 3 - Inclusion and Exclusion Criteria

**Concepts**	**Included**	**Excluded**
Concept one	People in custody (prison)<br>• Prison cell<br>• Health centre cell (including detox cell, cells assigned for people at increased risk of suicide placed on a Risk Intervention Team (RIT)<br>• Camera cell<br>• In transit in corrections vehicle<br>• People in custody in police stations under correctional services<br><br>People in custody (police)<br><br>• Holding cell<br>• In transit in police vehicles<br>• Dock (in police station)<br>• Court cells<br>• People that are in a police cell, have been police bail-refused and transferred to CSNSW custody awaiting court appearance (remaining in a police cell)	Detainees that do NOT refer to people in custody, including but not limited to:<br>• Immigration, military, facilities holding prisoners of war

Concept two	Prison<br><br>• Adult<br>• Juvenile Justice Centres (young people)<br>• Forensic psychiatric facilities<br><br>Police station<br><br>• Police watch house<br>• Lock-up	Studies that do NOT refer to custodial settings, including but not limited to:<br>• Child protection settings<br>• Non-forensic psychiatric hospitals<br>• External health services
Concept three	Knowledge/attitudes/perceptions (KAP) of custodial staff towards contactless monitoring in custodial settings, including but not limited to:<br><br>• Correctional officers<br>• Police officers<br>• Nurses	Studies that do NOT refer to KAP of custodial staff, including but not limited to:<br><br>• KAP of people in custody
Study types	• Systematic reviews and meta-analyses<br>• Other literature reviews published<br>• Relevant empirical research studies published including:<br>• Randomized Control trials (RCTs)/ non-RCTs<br>• Quasi-experimental (e.g. uncontrolled trials; pre/post-test designs)<br>Observational studies (e.g. cross-sectional surveys, cohort, case–control)<br>• Quantitative and qualitative (incl. qualitative only) mixed-design studies<br>• Grey literature, including conference abstracts, and dissertations	Literature in languages other than English.<br>Specific grey literature including:<br>• Newspaper articles, commentaries, editorials<br>All other not meeting inclusion criteria.

Supplementary 4 (S4)

Table 4: Data extraction plan

Category	Data to be extracted
Author (s)	List of authors
Year	Year of publication
Country (state)	Study country of origin and state if provided
Study design (if applicable)	Research question, study objectives/aims, study design, methods, publication type (i.e., academic/scientific paper, grey literature, press release, organisation report, etc)
Participant setting and sample	Setting – type of custodial settings (Prison, jail, police station, sentenced/remand, adult/juvenile, male/female facility), sample (number of participants)
Characteristics	Characteristics (age, gender, race/ethnicity)
Types of contactless monitoring technology	Types of technology/contactless sensing modalities being used (e.g. mmWave radar, image and non-image-based systems, vison based), vital signs being monitored (e.g. heart rate, blood pressure, respiratory rate, temperature, movement), other activities/behaviours being monitored (pacing, movement, bodily fluids, phone calls, visits, self-harm history)
Knowledge/attitudes/perceptions (KAP) of custodial staff	KAP of correctional officers, police officers, nurses.
Evaluation and outcome data	Evaluation of any trials of technology or outcome data for implemented technology.
