# Supplementary material for: Contactless monitoring to prevent self-harm and suicide in custodial settings: Protocol for a global scoping review
Source: BMJ Open. 2024 Oct 26;14(10):e087925. doi: 10.1136/bmjopen-2024-087925 (PMC11529512; doi:10.1136/bmjopen-2024-087925)
Supplement: Supplementary file 2 [file bmjopen-2024-087925.reviewer_comments.pdf]

PEER REVIEW HISTORY

BMJ Open publishes all reviews undertaken for accepted manuscripts. Reviewers are asked to complete a checklist review form and are provided with free text boxes to elaborate on their assessment. These free text comments are reproduced below.

ARTICLE DETAILS

Title (Provisional)

Contactless monitoring to prevent self-harm and suicide in custodial settings: protocol for a global scoping review

Authors

Bosworth, Dr Rebecca; Everett, Bronwyn; Breen, Paul; Klein, Jason; Psillakis, Eleni; Abbott, Penelope; Smith, Kirsty; Li, Wanqing; Anderson, Neil; Thakur, Chetan Singh; Borschmann, Rohan

---

VERSION 1 - REVIEW

**Reviewer**	**1**
**Name**	**Obegi, Joseph H**
**Affiliation**	**California Department of Corrections and Rehabilitation**
**Date**	**17-May-2024**
**COI**	**I have no competing interests.**

Correctional officials are frequently under intense pressure to reduce or eliminate suicides. This pressure often compels them to entertain solutions on the fringes of science in the hope that quick headway can be made (Hayes, 2013). Contactless monitoring of vital signs or movement exists near those fringes. Reviews of contactless monitoring would benefit from attending to several areas.

First, reviews should describe the developmental stage of contactless monitoring. Although researchers have shown that this type of real-time measurement tool, which includes technologies such as types of radar, camera imaging, and video imaging, can assess certain types of vital signs under specific conditions, many practical problems affecting accuracy exist (Khanam et al., 2019). The performance of these technologies (i.e., reliability, accuracy, utility) compared to traditional contact methods should also be described.

Secondly, reviews should describe the current scale of deployment of contactless monitoring in healthcare settings. This information will help readers gauge the level of acceptance and usage of contactless monitoring in mainstream healthcare settings. Adopting a technology for monitoring vital signs or behaviors that is not yet widely used in healthcare should give any correctional official pause. To my knowledge, contactless monitoring is still a rarity in everyday healthcare, with sensors in contact with the body remaining the norm.

Third, reviews should describe the limitations of scoping reviews, particularly their potential to confuse the existence of studies of some tool with the prevalence of the tool's use. A scoping review may well find

studies of contact monitoring in jails or prisons. However, this finding alone should not be taken to mean that the contactless monitoring of vital signs in jails or prisons is widespread or that the tool is bonafide. Similarly, studies showing that custodial staff report that contactless monitoring is acceptable should not be confused with evidence that the method is reliable or accurate.

Finally, reviews should not overstate the potential of contactless monitoring to alleviate privacy concerns. Some types of contactless monitoring rely on analyzing video feeds. Consequently, contactless monitoring may become a more serious threat to privacy and confidentiality than intermittent visual observation by custodial or healthcare staff. In addition, the technology's complexity requires more thoughtful informed consent practices.

Reviewer 2

Name Plugge, Emma

Affiliation University of Southampton Faculty of Medicine, Department of Primary Care, Population Sciences and Medical Education

Date 23-May-2024

COI None

---

1. Is the research question or study objective clearly defined?

Yes

2. Is the abstract accurate, balanced and complete?

I have marked no as there is a discrepancy between what is stated in the syntheized the evidence base regarding

3. Is the study design appropriate to answer the research question?

Yes, a scoping review is appropriate. However I have some concerns:

1. Lack of quality appraisal of included studies. I recognise that Arksey & O'Malley do not recommend this but their paper is now dated and scoping review methodology has since developed (and this is reflected in PRISMA ScR guidelines which include a section on QA). Most ScR now include quality assessment. The team should reflect carefully whether any robust conclusions can really be drawn from literature when they have not assessed the quality.

2. The inclusion of English language only literature. I appreciate that these technologies are likely to be found only in high-income countries, but the lack of inclusion of other languages might well mean that important information is lost. It will be important to ensure that the authors gather information on the number of papers excluded because of this criterion.

4. Are the methods described sufficiently to allow the study to be repeated?

Yes.

5. Are research ethics (e.g. participant consent, ethics approval) addressed appropriately?

Yes. These are not necessary.

6. Are the outcomes clearly defined?

N/A

7. If statistics are used are they appropriate and described fully?

N/A

8. Are the references up-to-date and appropriate?

Yes.

9. Do the results address the research question or objective?

N/A

10. Are they presented clearly?

N/A

11. Are the discussion and conclusions justified by the results

N/A

12. Are the study limitations discussed adequately?

N/A

13. Is the supplementary reporting complete (e.g. trial registration; funding details; CONSORT, STROBE or PRISMA checklist)?

Yes.

14. To the best of your knowledge is the paper free from concerns over publication ethics (e.g. plagiarism, redundant publication, undeclared conflicts of interest)?

Yes.

15. Is the standard of written English acceptable for publication?

Yes, it is very clear. Please re-read for typos. For example a ')' is missing, page 2, line 24.

Please also reconsider the use of the word 'contactless' which the authors seem to use interchangeably with non-invasive monitoring. The two are not the same.

General comments

This is likely to be an important review and I would urge the authors to consider QA of included studies.

Patient and public involvement: it's stated that a 'formerly incarcerated public partner, with lived experience of mental ill-health, is involved in the design, conduct, reporting, or dissemination plans of this research'. It's not clear whether this individual has experience of incarceration; such lived experience is very important here. Note also that another key PPI group are custodial staff, given the review's aim to examine 'the acceptability and feasibility of its application among custodial staff'. The extent to which they have been involved in this study is not clear.

**Reviewer**	**3**
**Name**	**Mahoney, Adam**
**Affiliation**	**Edinburgh Napier University, Psychology**
**Date**	**29-May-2024**
**COI**	**No competing interestes. Yes I consent.**

The proposal review has considerable merit and I wish you well in the eventual publication of your outcomes. I have spotted a typo on page 5 line 20, the word however is used twice. Similarly, I think it would read better if all of the authors are refered in the manuscript by their initals rather than a mix of full names and initals.

VERSION 1 - AUTHOR RESPONSE

Reviewer	Comment	Response	Page no.
Reviewer 1	First, reviews should describe the developmental stage of contactless monitoring. Although researchers have shown that this type of real-time measurement tool, which includes technologies such as types of radar, camera imaging, and video imaging, can assess certain types of vital signs under specific conditions, many practical problems affecting accuracy exist (Khanam et al., 2019).	Noted and attended to with thanks.<br><br>While non-invasive monitoring technology shows promise for addressing critical health concerns in custodial settings, its development stage must be considered. Despite advancements, contact-based sensors remain the norm in healthcare. Non-invasive monitoring is not yet standard practice, with limited literature on its effectiveness on acutely unwell or deteriorating patients (1). Validation against gold standard measurements in traditional settings is unclear (1). Both radar-based and camera-based techniques face challenges affecting accuracy and applicability (2). A review by Khanam et al. (2019) (2) on remote	p. 4

		monitoring of vital signs in diverse non-clinical and clinical scenarios using computer vision systems provides a thorough assessment of image-based monitoring and highlighted some deficiencies including (i) automatic selection of multiple regions of interest (ROIs), (ii) noise and motion artifact removal, (iii) simultaneous multi-person monitoring, (iv) long-distance detection, (v) multi-camera fusion, (vi) low lighting conditions, and (vii) the lack of publicly available datasets from realistic scenarios (2). Wireless video-based patient monitoring was thoroughly reviewed in a systematic review by Harford et al. (2017)(3), identifying several significant shortcomings including: (i) minimal testing or validation in clinical settings, (ii) a predominant focus on neonates rather than children or adults, and (iii) inadequate data for validation in laboratory settings, particularly concerning the duration of testing and the range of vital signs assessed in healthy participants. Radar-based technologies also encounter issues such as body movement interference and the lack of efficient and stable signal processing techniques capable of handling low sample	

		data (4). While Doppler radar has shown feasibility for vital sign monitoring in controlled environments, additional work is needed to improve signal quality analysis for better breathing and heart rate estimation (5). In prison settings, additional sources of motion like ceiling fans and water movement from sinks and toilet flushing further affect radar signal quality and increase false alarms (5, 6). Thus, challenges remain to widespread adoption in clinical settings and necessitate further research for widespread adoption.	
	The performance of these technologies (i.e., reliability, accuracy, utility) compared to traditional contact methods should also be described.	Noted and attended to with thanks.<br><br>Non-invasive monitoring technologies, while promising, often show reduced accuracy and reliability compared to traditional methods. However, studies have shown Doppler radar can match wearable device outputs within +/-5% for heart and respiratory rates (6). Gupta (2022) (7) reported 93.2%-100% accuracy for medical radar compared to contact-type ECGs and respiration belts. Camera-based measurements also perform well under ideal conditions but highlight performance variability (7, 8).	p. 4

	Secondly, reviews should describe the current scale of deployment of contactless monitoring in healthcare settings. This information will help readers gauge the level of acceptance and usage of contactless monitoring in mainstream healthcare settings.<br><br>Adopting a technology for monitoring vital signs or behaviors that is not yet widely used in healthcare should give any correctional official pause.<br><br>To my knowledge, contactless monitoring is still a rarity in everyday healthcare, with sensors in contact with the body remaining the norm.	Noted and attended to with thanks.<br><br>When considering the implementation of non-invasive monitoring technology, it's important to note its current deployment and acceptance in clinical settings. Some areas, like neonatal intensive care units, use camera imaging-based systems using imaging photoplethysmography (iPPG) for continuous monitoring, including heart rate, respiratory rate, skin temperature, and oxygen saturation (9, 10). iPPG has also been used for patients undergoing haemodialysis (11, 12). Trials at the Royal Melbourne Hospital involve radar imaging and thermal scanners for rapid assessment in elderly patients (13). A review by Grech (2024) (14) reported on 15 hospital-based studies on non-contact red-green-blue (RGB) camera-based heart rate and rhythm monitoring in adult clinical settings, including emergency departments, post-operative care units, general medical wards, and haemodialysis units. However, the review highlights ongoing challenges with patient movement, illumination, and technique standardization that	pp. 4-5

		must be overcome for widespread adoption (14).	
	Third, reviews should describe the limitations of scoping reviews, particularly their potential to confuse the existence of studies of some tool with the prevalence of the tool's use.<br><br>A scoping review may well find studies of contact monitoring in jails or prisons. However, this finding alone should not be taken to mean that the contactless monitoring of vital signs in jails or prisons is widespread or that the tool is bonafide.	Noted and attended to with thanks.	p. 5
	Similarly, studies showing that custodial staff report that contactless monitoring is acceptable should not be confused with evidence that the method is reliable or accurate.	Noted and attended to with thanks.<br><br>Limited literature exists on staff perceptions of non-contact monitoring. Ede et al. (2021) explored intensive care unit staff expectations, finding the concept acceptable with perceived usability benefits for both patients and staff (15). Non-contact monitoring may offer a sustainable solution, yet staff need to be comfortable and familiar with the system and able to troubleshoot issues independently (15). Despite this, perceived acceptability does not equate to proven reliability or accuracy of the technology.	p. 5

	Finally, reviews should not overstate the potential of contactless monitoring to alleviate privacy concerns.<br><br>Some types of contactless monitoring rely on analyzing video feeds. Consequently, contactless monitoring may become a more serious threat to privacy and confidentiality than intermittent visual observation by custodial or healthcare staff.<br><br>In addition, the technology's complexity requires more thoughtful informed consent practices.	Noted and attended to with thanks.<br><br>Despite the promise of this technology, the continuous collection of sensor data in healthcare settings presents significant ethical concerns about privacy, data management, bias, fairness, and informed consent (18). Therefore, addressing these issues is crucial to identify and mitigate potential harms, ensuring transparency and accountability and build trustworthy and ethically sound systems (18).	p. 5
Reviewer 2	Is the abstract accurate, balanced and complete?<br>I have marked no as there is a discrepancy between what is stated in the syntheized the evidence base regarding	As the comment appears to be incomplete, we have done our best to identify the reviewer's concern.<br><br>In response, we have amended the abstract to include the following; 'however, no reviews to date have synthesized the evidence base, in the custodial context, ….<br><br>We have also amended this in the body: However, no reviews to date have synthesized the evidence base, in the custodial context, regarding the feasibility and acceptability from the perspective of end users, including people detained in custodial settings, custodial officers, and healthcare staff, and on the extent to which contactless	p. 2, 5

		monitoring has been implemented in custodial settings.	
	Lack of quality appraisal of included studies. I recognise that Arksey & O'Malley do not recommend this but their paper is now dated and scoping review methodology has since developed (and this is reflected in PRISMA ScR guidelines which include a section on QA). Most ScR now include quality assessment. The team should reflect carefully whether any robust conclusions can really be drawn from literature when they have not assessed the quality.	The authors have added:<br><br>The Joanna Briggs Institute Critical Appraisal Checklist will be used to assess the methodological quality of all primary research publications by evaluating the extent to which they addressed the possibility of bias in nine areas of study design, conduct, and analysis.	p. 5
	The inclusion of English language only literature. I appreciate that these technologies are likely to be found only in high-income countries, but the lack of inclusion of other languages might well mean that important information is lost. It will be important to ensure that the authors gather information on the number of papers excluded because of this criterion.	The authors acknowledge this is a limitation of the study on p. 2.<br><br>All results in languages other than English were excluded at the abstract, title stage, therefore, exclusion reasons were not noted.<br><br>Only when the study is excluded in the full text would the reason for exclusion be noted. Therefore, it is not possible to gather this information on the number excluded due to language.	p. 2
	Please re-read for typos. For example a ')' is missing, page 2, line 24.	Amended typo with thanks.	p. 2
	Please also reconsider the use of the word 'contactless' which the authors seem to use	The use of the word 'non-invasive' has been reconsidered and replaced with	

	interchangeably with non-invasive monitoring. The two are not the same.	'contactless' throughout.	
	Patient and public involvement: it's stated that a 'formerly incarcerated public partner, with lived experience of mental ill-health, is involved in the design, conduct, reporting, or dissemination plans of this research'. It's not clear whether this individual has experience of incarceration; such lived experience is very important here.	Further clarity provided around the lived experience of incarceration.	p. 7
	Note also that another key PPI group are custodial staff, given the review's aim to examine 'the acceptability and feasibility of its application among custodial staff'. The extent to which they have been involved in this study is not clear.	Further clarity provided regarding the involvement of custodial staff has been provided.<br><br>The authors acknowledge that involvement of custodial staff in the review would also be important, however, this was not possible at the time.<br><br>This has been added to the limitation section.<br><br>A limitation of this study is that correctional officers do not comprise the team of stakeholders.	p. 2
Reviewer 3	I have spotted a typo on page 5 line 20, the word however is used twice. Similarly, I think it would read better if all of the authors are referred in the manuscript by their initials rather than a mix	Removed typo with thanks.<br><br>The rationale for using full text and initials is due to the first and last author having the same initials, RB, and the need to differentiate	p. 5

	of full names and initials.	between the two. Therefore, this has remained unchanged.	

References

1. Chan PY, McNeil J, Nguyen T, Ryan N, Hopper I. Novel wearable and contactless monitoring devices to identify deteriorating patients in the clinical setting: a systematic review protocol. Systematic Reviews. 2020;9:1-8.
2. Khanam F-T-Z, Al-Naji A, Chahl J. Remote monitoring of vital signs in diverse non-clinical and clinical scenarios using computer vision systems: A review. Applied Sciences. 2019;9(20):4474.
3. Harford M, Catherall J, Gerry S, Young JD, Watkinson P. Availability and performance of image-based, non-contact methods of monitoring heart rate, blood pressure, respiratory rate, and oxygen saturation: a systematic review. Physiological measurement. 2019;40(6):06TR1.
4. Rehouma H, Noumeir R, Essouri S, Jouvet P. Advancements in methods and camera-based sensors for the quantification of respiration. Sensors. 2020;20(24):7252.
5. Tworzydlo P, Chan A, editors. Spectral Analysis of Respiratory and Cardiac Signals Using Doppler Radar. World Congress on Medical Physics and Biomedical Engineering, June 7-12, 2015, Toronto, Canada; 2015: Springer.
6. Gagnon A, editor Field trial results using a novel integration of unique millimeterwave Doppler radar for high performance non-obtrusive life sign (breathing and heart beating) monitoring of high suicide risk prisoner in observation cell. 2016 IEEE International Carnahan Conference on Security Technology (ICCST); 2016: IEEE.
7. Gupta K, Srinivas M, Soumya J, Pandey OJ, Cenkeramaddi LR. Automatic contact-less monitoring of breathing rate and heart rate utilizing the fusion of mmWave radar and camera steering system. IEEE Sensors Journal. 2022;22(22):22179-91.
8. Malmberg S, Khan T, Gunnarsson R, Jacobsson G, Sundvall P-D. Remote investigation and assessment of vital signs (RIA-VS)—proof of concept for contactless estimation of blood pressure, pulse, respiratory rate, and oxygen saturation in patients with suspicion of COVID-19. Infectious Diseases. 2022;54(9):677-86.
9. Villarroel M, Guazzi A, Jorge J, Davis S, Watkinson P, Green G, et al. Continuous non-contact vital sign monitoring in neonatal intensive care unit. Healthcare technology letters. 2014;1(3):87-91.
10. Klaessens JH, Van Den Born M, Van Der Veen A, Sikkens-Van De Kraats J, van den Dungen FA, Verdaasdonk RM, editors. Development of a baby friendly non-contact method for measuring vital signs: first results of clinical measurements in an open incubator at a neonatal intensive care unit. Advanced biomedical and clinical diagnostic systems XII; 2014: SPIE.
11. Villarroel M, Jorge J, Pugh C, Tarassenko L, editors. Non-contact vital sign monitoring in the clinic. 2017 12th IEEE international conference on automatic face & gesture recognition (FG 2017); 2017: IEEE.
12. Tarassenko L, Villarroel M, Guazzi A, Jorge J, Clifton D, Pugh C. Non-contact video-based vital sign monitoring using ambient light and auto-regressive models. Physiological measurement. 2014;35(5):807.
13. The Royal Melbourne Hospital. Remote monitoring device trial underway at the RMH 2023 [Available from: https://www.thermh.org.au/news/remote-monitoring-device-trial-underway-at-the-rmh.
14. Grech N, Agius JC, Sciberras S, Micallef N, Camilleri K, Falzon O. Non-invasive Vital Signs Monitoring in the Adult Population in Clinical Settings–Current State of the Art and Beyond. XJENZA Online. 2024;12(1):0-.
15. Ede J, Vollam S, Darbyshire JL, Gibson O, Tarassenko L, Watkinson P. Non-contact vital sign monitoring of patients in an intensive care unit: A human factors analysis of staff expectations. Applied Ergonomics. 2021;90:103149.

**** Response to Reviewer - BMJ Open_CoverLetter_Revisions_20240821.docx ****

VERSION 2 - REVIEW

**Reviewer**	**1**
**Name**	**Obegi, Joseph H**
**Affiliation**	**California Department of Corrections and Rehabilitation**
**Date**	**05-Sep-2024**
**COI**	**None.**

I have read the authors' resubmission and appreciate their receptiveness to the concerns raised. I believe the revised manuscript gives a more balanced presentation of the current technical state of contactless monitoring as well as the practical challenges involved in its use in correctional settings. I would like to offer two minor points of feedback:

First, on page 3, the authors refer to suicide risk assessment as if it were a type of monitoring in the same class as visual observation. A difference exists between risk assessments and continuous or near-continuous monitoring of a patient's immediate safety and welfare. By completing clinically indicated suicide risk assessments, clinicians are re-evaluating the patient's risk of future suicidal behavior, not monitoring suicide risk in the sense of continuous or frequent observation. Rather, suicide risk assessment is a point-in-time tool used to determine whether enhanced monitoring (i.e., continuous observation or 15-minute checks) is indicated.

Second, on page 4, change the contraction "It's" to "It is."

**Reviewer**	**2**
**Name**	**Plugge, Emma**
**Affiliation**	**University of Southampton Faculty of Medicine, Department of Primary Care, Population Sciences and Medical Education**
**Date**	**24-Sep-2024**
**COI**	**None**

The authors have addressed the concerns raised by the reviewers

VERSION 2 - AUTHOR RESPONSE

Reviewer	Comment	Response	Page no.
Reviewer 1	First, on page 3, the authors refer to suicide risk assessment as if it were a type of monitoring in the same class as visual observaWon. A difference exists between risk assessments and conWnuous or near-conWnuous monitoring of a paWent's immediate safety and welfare. By compleWng clinically indicated suicide risk assessments, clinicians are re-evaluaWng the paWent's risk of future suicidal behavior, not monitoring suicide risk in the sense of conWnuous or frequent observaWon. Rather, suicide risk assessment is a point-in-Wme tool used to determine whether enhanced monitoring (i.e., conWnuous observaWon or 15-minute checks) is indicated.	Noted and aXended to with thanks.<br><br>TradiWonal monitoring methods include risk assessments and visual observaWons, both physically and remotely, yet key differences exist. Risk assessments are informed by both clinical intuiWon and screening on entry into custody and as circumstances or condiWons change (1-3), assessing for signs of intoxicaWon and/or withdrawal, consciousness levels, head injuries, substance concealment, and self-harm or non-fatal suicide aXempt history (4). Risk assessments are on-going and refer to structured, point-in-Wme evaluaWons used to determine whether increased monitoring such as conWnuous	p. 3

		observaWon or checks at specific intervals are indicated (1). Risk assessments are also used to determine if monitoring can be decreased indicated by the individual's clinical condiWon and future risk factors for suicide or self-harm (5). Visual observaWons refer to watching for observable changes that may indicate risk and may include physically checking for signs of life via posiWonal changes, aXempts to rouse when sleeping, or movement in the rise and fall of chest (3, 4, 6), either from the cell door or remotely via CCTV video surveillance (1, 2, 4).	
	Second, on page 4, change the contracWon "It's" to "It is."	Noted and aXended to with thanks.	p. 4

References

1. Metropolitan Police. Custody tool kit 2022 [Available from: https://www.met.police.uk/SysSiteAssets/foi-media/metropolitan-police/disclosure_2022/may_2022/custody-policy-toolkit-detainees-policy.pdf.
2. OHice of the Police and Crime Commissioner Devon and Cornwall. High-tech cameras will save lives in Devon and Cornwall Police custody cells 2023 [Available from: https://www.devonandcornwall-pcc.gov.uk/news-and-blog/multimedia-hub/high- tech-cameras-will-save-lives-in-devon-and-cornwall-police-custody-cells/.
3. Australian Federal Police. AFP National Guideline on persons in custody and police custodial facilities 2012 [Available from: https://www.afp.gov.au/sites/default/files/PDF/IPS/AFP%20National%20Guide%20on% 20Persons%20in%20Custody%20and%20Police%20Custodial%20Facilities% 20and%2 0People%20in%20Custody%2010MAY2012.pdf.

4. Independent OHice for Police Conduct. Annual deaths during or following police contact report - 2022/23 2023 [Available from: https://www.policeconduct.gov.uk/publications/annual-deaths-during-or-following- police-contact-report-202223.
5. Justice Health and Forensic Mental Health Network. Clinical Care of People Who May Be Suicidal (ImpG) 2022 [Available from: https://disability.royalcommission.gov.au/system/files/exhibit/NSW.0074.0014.0001.pd f.
6. PoliceOracle. Cameras monitoring detainees heartrate 'can make custody safer' 2023 [Available from: https://touch.policeoracle.com/news/article.cfm?id=92349.
